# Differential prognostic value of MYC immunohistochemistry in subtypes of papillary renal cell carcinoma

**DOI:** 10.1038/s41598-017-16144-4

**Published:** 2017-11-27

**Authors:** Julia Bellut, Simone Bertz, Elke Nolte, Christine Stöhr, Iris Polifka, Verena Lieb, Edwin Herrmann, Rudolf Jung, Arndt Hartmann, Bernd Wullich, Helge Taubert, Sven Wach

**Affiliations:** 1 Department of Urology and Pediatric Urology, University Hospital Erlangen, FAU Erlangen-Nürnberg, Erlangen, Germany; 2 Institute of Pathology, University Hospital Erlangen, FAU Erlangen-Nürnberg, Erlangen, Germany; 3 University Hospital Münster, Münster, Germany

## Abstract

The histomorphological subtyping of papillary renal cell carcinomas (pRCCs) has improved the predictions of patients’ long-term survival. Based on our previous results, we hypothesized that the MYC proto-oncogene would show differential expression in pRCC subtypes. Using a multi-institutional cohort of 204 pRCC patients we assessed the additional value of the immunohistochemical markers MYC, MINA53, and Ki67 in predicting patient’s long-term survival. The clinical endpoints were overall survival (OS) and cancer-specific survival (CSS). Nomograms were constructed to predict each patient’s risk of death (OS). The incorporation of the MYC staining patterns allowed the stratification of pRCC type 1 patients into better and worse prognostic groups. None of the patients with pRCC type 1 tumors and favorable MYC staining patterns died from tumor-related causes. This prognostic value was independent of the patient’s age at surgery, the pathological tumor stage and presence of lymph node invasion. we could show that the immunohistochemical assessment of MYC and the histomorphological subtyping of pRCC stratifies pRCC type 1 tumors with regard to OS and CSS. The determination of the histomorphologic pRCC subtype in combination with the MYC immunohistochemical staining patterns allows a more accurate prediction of patients’ individual risk of death.

## Introduction

With an estimated 338,000 new cases in 2012, renal cell carcinoma (RCC) accounts for 2.4% of all cancer cases worldwide^1^. According to the WHO classification for tumors of the urinary system^2^, RCC includes multiple subtypes. In addition to the conventional clear cell RCC, papillary RCC (pRCC) is the second most common subtype, which represents 10–15% of all RCCs^3,4^. Based on their histomorphological characteristics, pRCCs can further be sub-classified into two distinct subtypes^5^. Independent studies have demonstrated that type 2 pRCCs (pRCC2) are associated with a worse clinical outcome compared to type 1 pRCCs (pRCC1)^5–7^. While type 2 pRCCs contain multiple molecular subtypes, the type 1 pRCCs are a very homogeneous group^8^. New markers would be helpful in identifying aggressive cases within type 1 pRCCs.

MicroRNAs (miRNAs) are small, non-coding RNA molecules of approximately 19–25 nucleotides. When complexed with argonaute proteins within the RNA-induced silencing complex, miRNAs contribute to post-transcriptional gene silencing^9^. The miRNA expression patterns are highly specific and are able to discriminate between different tumor entities^10^, and even between different RCC subtypes^11^. We have previously established the miRNA expression profiles of pRCCs, with a particular focus on identifying miRNAs that distinguish between pRCC subtypes 1 and 2 (ref.^12^). We identified miRNAs, *miR-210* and *let-7c*, which were able to discriminate between the two distinct pRCC subtypes with high accuracy. To gain insight into the molecular consequences of deregulated miRNA expression, we performed a gene set enrichment analysis and found that 10 genes involved in the Jak-STAT pathway are potential targets of the deregulated miRNAs *miR-210* and *let-7c*, including the v-myc myelocytomatosis viral oncogene homolog (*MYC*)^12^. *MYC* is a validated target of *let-7c* ^13,14^. MYC activation has been described in high grade pRCCs^15^. In transgenic mouse models, ectopic MYC expression was sufficient to induce RCCs that, depending on the promoter driving ectopic MYC expression, resemble different RCC entities. MYC under the control of the γ-glutamyl transferase promoter induced RCCs that resembled human collecting duct carcinomas^16^, while MYC under control of the Cadherin-16 promoter induced high-grade papillary tumors^17^. One target that is directly regulated by MYC is the myc-induced nuclear antigen, MINA53. In a series of 34 pRCCs, MINA53 is one of the genes that exhibited prominent overexpression in pRCC type 2 tumors^18^. Moreover, in a patient series consisting predominantly of clear cell RCCs, it has been demonstrated that the MINA53 immunohistochemical staining patterns correlated with the Ki67 labeling index and the patients’ survival^19^.

The aim of the present study was to assess the additional value of the immunohistochemical markers MYC, MINA53, and Ki67 in predicting patient’s long-term survival.

## Results

### Clinico-pathological characteristics

**Figure 1:**
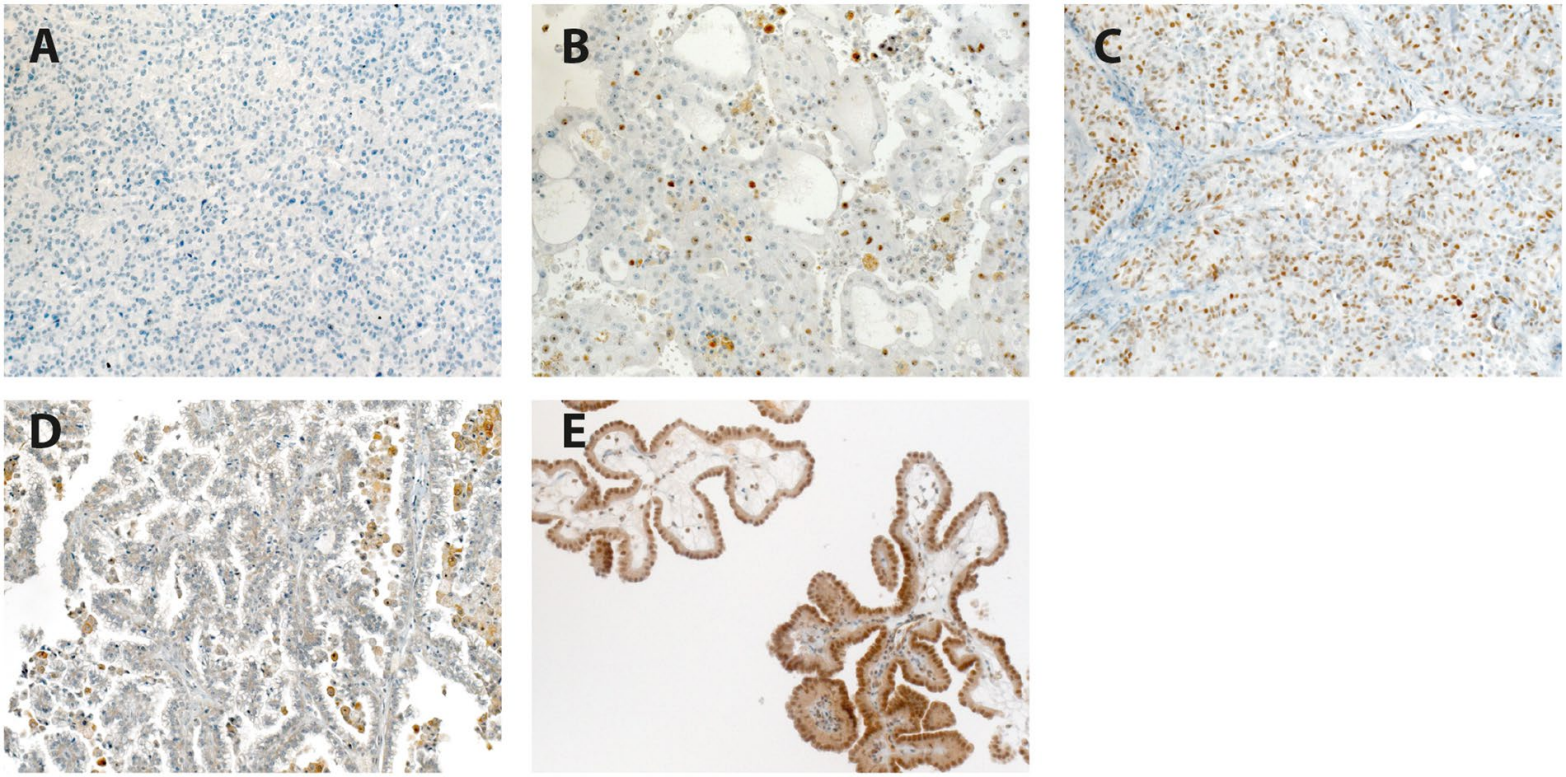
Immunohistochemical evaluation. Representative pRCC tissue sections with absent MYC staining (**A**), intermediate MYC staining (**B**) and strong MYC staining (**C**) are shown. Representative pRCC tissue sections with negative MINA53 staining (**D**) and positive MINA53 staining (**E**) are shown. Final magnification 200x.

A total of 204 patients with papillary RCC were included in this study: 113 patients with pRCC type 1, 39 patients with pRCC type 2, and 34 patients with mixed-histology pRCCs. For 18 patients, the histologic pRCC subtype remained unknown. A total of 42 patients died during the observation period, and 26 of these patients died from tumor-related causes. The median follow-up period was 35.5 months (range 1–172 months). The clinico-pathological characteristics of the patient cohort are shown in Table 1. To analyze the MINA53 immunohistochemistry, we classified the tissue spots either as negative or positive. MINA53 exhibited a homogeneous nuclear staining. For the Ki67 labeling index, we applied a cutoff of 5% of stained tumor cells. To interpret the MYC staining patterns, we established a scoring system of negative, intermediate and strong staining. A lack of staining was scored as negative, a tumor sample was regarded as strongly stained if at least 50% of the tumor cells exhibited strong MYC staining, and the remaining samples were regarded as intermediate. We were able to detect distinct levels of MYC expression, ranging from negative (33.3%) to intermediate (55.9%) to strong staining (10.8%). The distribution of the staining patterns between the pRCC subtypes is shown in Table 1. Representative pictures of the MYC and MINA53 immunohistochemistry are shown in Fig. 1.

**Table 1:** Patient’s characteristics.

	pRCC1 (N = 113)	pRCC2 (N = 39)	Mixed (N = 34)	P
Age at surgery; median (IQR)	63 (54–69)	66 (60.5–71.25)	72 (58.75–77.75)	0.001
Gender; N (%)				0.729
Female (N = 39)	22 (19.5)	10 (25.6)	7 (20.6)	
Male (N = 146)	90 (79.6)	29 (74.4)	27 (70.4)	
n.a. (N = 1)	1 (0.9)	0	0	
pT stage; N (%)				<0.001
pT1 (N = 109)	78 (69.0)	17 (43.6)	14 (41.2)	
pT2 (N = 39)	27 (23.9)	6 (15.4)	6 (17.7)	
pT3 (N = 37	8 (7.1)	16 (41.0)	13 (38.2)	
pT4 (N = 1)	0	0	1 (2.9)	
pN; N(%)				<0.001
pN0 (N = 157)	105 (92.9)	26 (66.6)	26 (76.5)	
pN1 (N = 8)	1 (0.9)	4 (10.3)	3 (8.8)	
pN2 (N = 14)	3 (2.7)	6 (15.4)	5 (14.7)	
n.a. (N = 7)	4 (3.5)	3 (7.7)	0	
pM; N (%)				<0.001
M0 (N = 165)	107 (94.7)	28 (71.8)	30 (88.2)	
M1 (N = 17)	4 (3.5)	9 (23.1)	4 (11.8)	
n.a. (N = 4)	2 (1.8)	2 (5.1)	0	
Grade; N (%)				<0.001
G1 (N = 48)	35 (31.0)	5 (12.8)	8 (23.5)	
G2 (N = 110)	70 (62.0)	23 (59.0)	17 (50.0)	
G3 (N = 22)	4 (3.5)	9 (23.1)	9 (26.5)	
n.a. (N = 6)	4 (3.5)	2 (5.1)	0	
Status OS; N (%)				<0.001
Alive (N = 139)	97 (86.0)	25 (64.1)	17 (50.0)	
Deceased (N = 42)	14 (12.2)	13 (33.3)	15 (44.1)	
n.a. (N = 5)	2 (1.8)	1 (2.6)	2 (5.9)	
Status CSS; N (%)				<0.001
Other (N = 155)	106 (94.0)	29 (74.3)	20 (58.8)	
Cancer-specific death (N = 26)	5 (4.2)	9 (23.1)	12 (35.3)	
n.a. (N = 5)	2 (1.8)	1 (2.6)	2 (5.9)	
MINA IHC; N (%)				0.015
Negative (N = 122)	73 (64.6)	32 (82.1)	17 (50.0)	
Positive (N = 64)	40 (35.4)	7 (17.9)	17 (50.0)	
MYC IHC; N (%)				0.785
Negative (N = 62)	38 (33.6)	12 (30.8)	12 (35.3)	
Intermediate (N = 104)	63 (55.8)	21 (53.8)	20 (58.5)	
Strong (N = 20)	12 (10.6)	6 (15.4)	2 (5.9)	
Ki67 IHC; N (%)				0.004
<5% (N = 116)	81 (71.7)	18 (46.2)	17 (50.0)	
≥5% (N = 65)	29 (25.6)	19 (48.7)	17 (50.0)	
n.a. (N = 5)	3 (2.7)	2 (5.1)	0	

IQR, interquartile range; OS, overall survival; CSS, cancer-specific survival; n.a., not available.

Neither the MYC nor MINA53 staining patterns were associated with the clinico-pathological parameters of age at surgery, gender, pT, pN, pM, tumor grade or patient’s survival (Chi-squared test; Supplementary Tables 1 and 2). A high Ki67 labeling index, with ≥5% of stained tumor cells, was associated with a higher pT stage, the occurrence of lymph node invasion, distant metastases, and a higher tumor grade (P < 0.05; Supplementary Table 3).

### Predictors of patients’ survival

**Figure 2:**
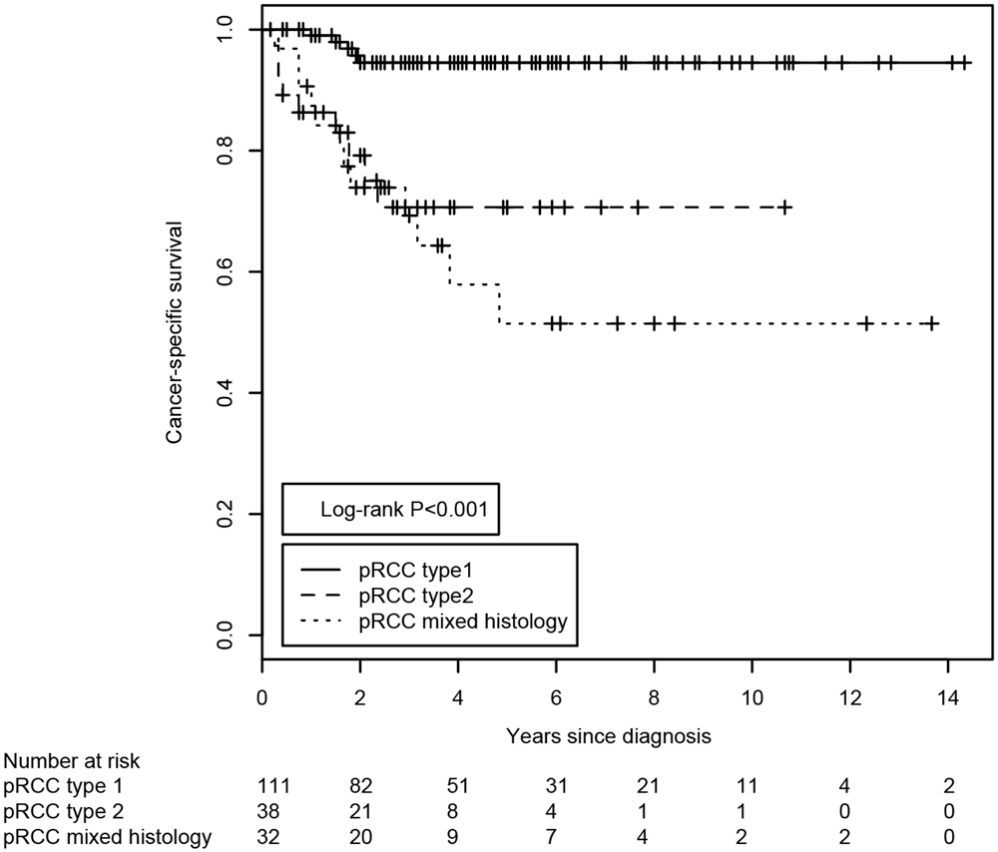
Kaplan-Meier estimates of the cancer-specific survival of patients with pRCC stratified according to the histological classification of the tumor. The P-value was derived from the log-rank test. The number of subjects at risk at the displayed 2-year intervals is indicated below the Kaplan-Meier graph.

Next, we examined the impact of the histopathological factors on the overall (OS) and cancer-specific survival (CSS) of pRCC patients. As expected, the histological subtype of the pRCC tumors was a major predictor of CSS (Fig. 2).

Patients whose tumors exhibited a mixed type 1/type 2 histology had an even worse CSS, with a mean survival of 98.2 months, compared to patients with type 2 (120.2 months) or type 1 pRCCs (156.1 months; log-rank p < 0.001). Regarding OS, patients with mixed-histology pRCCs had a mean survival of 85.5 months compared to patients with type 2 (93.1 months) or type 1 pRCCs (140.7 months; log-rank p < 0.001; Table 2 and Supplementary Fig. 1). Among the three immunohistochemical markers (MYC, MINA53 and Ki67), only the Ki67 labeling index was associated with CSS (Table 2 and Supplementary Fig. 7).

**Table 2:** Kaplan-Meier estimates of overall and cancer-specific survival.

	Overall survival				Cancer-specific survival			
	Restricted mean survival (months)	SD	Upper limit	P	Restricted mean survival (months)	SD	Upper limit	P
pRCC subtype				<0.001				<0.001
pRCC 1	140.7	5.78	164		156.1	3.4	164	
pRCC2	93.1	15.55	164		120.2	12.6	164	
Mixed	85.3	13.95	164		98.2	14.5	164	
MYC staining				0.625				0.395
Negative	124	8.94	164		142	6.9	164	
Intermediate	115	7.56	164		133	6.7	164	
Strong	104	20.11	164		150	13.2	164	
MYC staining				0.41				0.379
Negative/strong	121	8.56	163		144	6	163	
Intermediate	115	7.16	163		133	6.3	163	
MINA53 staining				0.304				0.243
Negative	126	7.29	170		149	5.3	170	
Positive	117	9.94	107		134	9.1	170	
Ki67 index				0.08				<0.001
<5%	126	6.29	168		152	4.6	168	
≥5%	104	11.9	168		119	10.3	168	
pRCC subtype + MYC				<0.001				<0.001
pRCC1 MYC negative/strong	135.1	4.07	141		141	0	141	
pRCC1 MYC intermediate	110.6	7.7	141		129	5.1	141	
pRCC2 MYC negative/strong	72.7	19.49	141		108	14.2	141	
pRCC2 MYC intermediate	91	14.71	141		103	14.3	141	
pRCC subtype + MINA53				0.003				<0.001
pRCC1 MINA53 negative	131.1	6.17	148		141.8	3.8	148	
pRCC1 MINA53 positive	123.5	8.49	148		141.1	5.1	148	
pRCC2 MINA53 negative	86.5	15.7	148		118.2	11.1	148	
pRCC2 MINA53 positive	86.2	25.48	148		86.2	25.48	148	
pRCC subtype + Ki67 index				<0.001				<0.001
pRCC1 Ki67 <5%	94.3	4.18	111		105.6	2.6	111	
pRCC1 Ki67 ≥5%	106.4	4.46	111		106.4	4.5	111	
pRCC2 Ki67 <5%	74.2	12.37	111		97.6	8.9	111	
pRCC2 Ki67 ≥5%	55.8	13.46	111		64.4	13.4	111	

SD, standard deviation.

Regarding the CSS of pRCC patients stratified by their MYC staining patterns, we observed that patients with intermediate MYC staining in their tumors exhibited the worst prognosis, while patients with negative or strong MYC staining had a tendency towards a better long-term survival. Although this was not significant (Table 2), patients with negative MYC staining had a 9-month advantage and patients with strong MYC staining had a 17-month advantage over intermediate MYC staining in estimated mean survival. Likewise, this difference was not significant in pair-wise comparisons between negative vs. intermediate or intermediate vs. strong. This somewhat counterintuitive result might be explained by the fact, that while low levels of deregulated MYC are able to drive oncogenesis, a high overexpression of MYC is able to activate ARF/p53 tumor suppressor pathway^20^. Moreover, when regarding the actual numbers of cancer-specific death events, we noted that only one of 22 patients (4.5%) with strong MYC staining suffered a cancer-related death, compared to 9 of 67 (13.4%) cases with absent MYC staining and 18 of 110 (16.4%) cases with intermediate MYC staining. Therefore, we decided to combine the groups with negative and strong MYC staining and regarded intermediate MYC staining as an adverse factor for patients’ survival.

**Figure 3:**
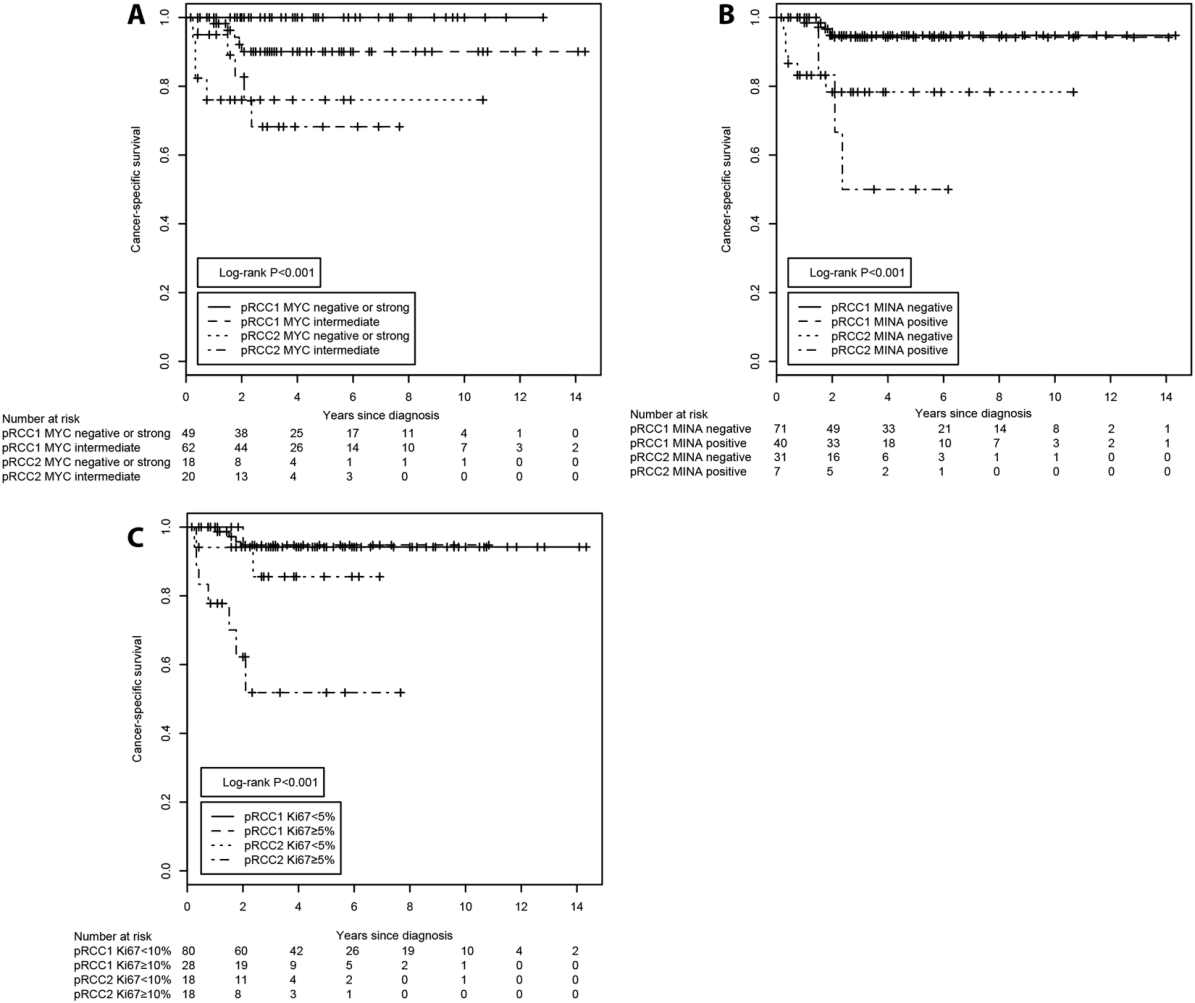
Kaplan-Meier estimates of the cancer-specific survival of patients with pRCC stratified according to the combination of histological classification and immunohistochemical staining patterns. The histomorphological subtype was combined with the (**A**) MYC staining patterns, (**B**) MINA53 staining patterns or (**C**) Ki67 labeling index. The P-values were derived from the log-rank test. The number of subjects at risk at the displayed 2-year intervals is indicated below the Kaplan-Meier graph.

Next, we combined the immunohistochemical staining patterns of MYC, MINA53 and Ki67 with the histomorphological pRCC tumor subtype (Fig. 3). We discovered that only MYC, but not the MINA53 or Ki67 staining patterns, was able to further sub-stratify the cohort of pRCC type 1 tumors. None of the patients with pRCC type 1 tumors that displayed negative or strong MYC staining patterns died of tumor-related causes during the complete observation period (Fig. 3A). Additional Kaplan-Meier analyses of OS and CSS are shown in Supplementary Figs 1–10.

To estimate the associated relative risks, we first performed univariate Cox’s proportional hazard regression analyses. The histomorphological subtype was significantly associated with an increased risk of death or cancer-specific death, with type 2 pRCCs conferring a 6.8-fold risk and pRCC tumors with a mixed morphology exhibiting a 9.2-fold risk of cancer-specific death in relation to type 1 pRCCs (p < 0.001; Table 3). Regarding OS, a pRCC type 2 histology was associated with a 3.8-fold risk and pRCC tumors with a mixed morphology were associated with a 4.2-fold risk of death (p < 0.001, Table 3). Likewise, an elevated Ki67 labeling index of ≥5% was associated with CSS, resulting in a 3.4-fold increased risk of cancer-specific death (p = 0.002; Table 3). As before, only the MYC staining patterns were able to sub-stratify the pRCC type 1 tumors. In OS, pRCC type 1 tumors with intermediate MYC staining were associated with a 5.3-fold increased risk of death compared to pRCC type 1 tumors with negative/strong MYC staining (p = 0.028; Table 3). For pRCC type 2 tumors, the MYC, MINA53 and Ki67 staining patterns were not associated with differences in OS (overlapping confidence intervals; Table 3

**Table 3:** Analysis of the patients’ overall and cancer-specific survival using Cox’s proportional hazard ratio.

Univariate analyses				
Parameter	Overall survival		Cancer-specific survival	
	Relative risk (95% CI)	P	Relative risk (95% CI)	P
pRCC subtype				
pRCC1	Reference		Reference	
pRCC2	3.795 (1.779–8.095)	<0.001	6.839 (2.288–20.440)	<0.001
Mixed	4.224 (2.038–8.756)	<0.001	9.187 (3.234–26.100)	<0.001
MYC staining				
Negative	Reference		Reference	
Intermediate	1.316 (0.704–2.457)	0.39	1.283 (0.576–2.858)	0.542
Strong	0.954 (0.316–2.878)	0.933	0.360 (0.047–2.904)	0.343
MYC staining				
Negative/strong	Reference		Reference	
Intermediate	1.282 (0.709–2.318)	0.412	1.424 (0.644–3.149)	0.383
MINA53 staining				
Negative	Reference		Reference	
Positive	1.352 (0.760–2.204)	0.305	1.551 (0.738–3.262)	0.247
Ki67 index				
<5%	Reference		Reference	
≥5%	1.664 (0.936–2.959)	0.083	3.395 (1.587–7.262)	0.002
pRCC subtype + MYC				
pRCC1 MYC negative/strong	Reference		Reference	
pRCC1 MYC intermediate	5.337 (1.194–23.860)	0.028	n.c.^b^	
pRCC2 MYC negative/strong	14.300 (2.875–71.180)	0.001	n.c.^b^	
pRCC2 MYC intermediate	11.226 (2.325–54.210)	0.003	n.c.^b^	
pRCC subtype + MINA53				
pRCC1 MINA53 negative	Reference		Reference	
pRCC1 MINA53 positive	1.665 (0.580–4.721)	0.347	1.095 (0.183–6.555)	0.921
pRCC2 MINA53 negative	4.624 (1.753–12.198)	0.002	5.571 (1.434–22.983)	0.013
pRCC2 MINA53 positive	4.951 (1.278–19.222)	0.021	10.300 (2.078–51.045)	0.004
pRCC subtype + Ki67 index				
pRCC1 Ki67 <5%	Reference		Reference	
pRCC1 Ki67 ≥5%	0.261 (0.034–1.998)	0.196	0.778 (0.087–6.956)	0.822
pRCC2 Ki67 <5%	2.227 (0.791–6.267)	0.129	2.604 (0.477–14.220)	0.269
pRCC2 Ki67 ≥5%	5.148 (2.096–12.643)	0.001	11.533 (3.340–39.821)	<0.001
**Multivariate analysis** ^a^				
		Overall survival	Cancer-specific survival	
Age at surgery	1.05	0.026	0.908	0.737
pT stage				
pT1	Reference		Reference	
pT2	0.738 (0.207–2.626)	0.638	1.113 (0.095–13.039)	0.932
pT3	5.609 (1.996–15.764)	0.001	14.350 (2.788–73.892)	0.001
pT4	n.a.		n.a.	
Lymph node invasion				
pN0	Reference		Reference	
pN1	11.661 (1.127–120.653)	0.039	9.341 (0.795–109.779)	0.076
pN2	20.425 (5.422–76.938)	<0.001	10.810 (2.481–47.114)	0.002
pRCC subtype + MYC				
pRCC1 MYC negative/strong	Reference		Reference	
pRCC1 MYC intermediate	6.371 (1.289–31.504)	0.023	n.c.^b^	
pRCC2 MYC negative/strong	12.782 (2.445–66.839)	0.003	n.c.^b^	
pRCC2 MYC intermediate	3.631 (0.694–18.995)	0.127	n.c.^b^	

^a^A multivariate analysis was only performed for the combination of the pRCC subtype and MYC because it had the potential to sub-stratify pRCC type 1 tumors. ^b^The relative risks were not calculated because no cancer-specific death occurred in the reference category. CI, confidence interval.

).

Therefore, we analyzed the combination of histomorphology and MYC staining patterns using a multivariate model and adjusted this model to the patient’s age at surgery, pathological tumor stage and presence of lymph node invasion. Here, the combination of the pRCC subtype and MYC staining patterns emerged as an independent prognostic parameter for the patient’s OS. Patients with pRCC type 1 tumors that exhibited an intermediate MYC staining intensity had a 6.4-fold increased risk of death (p = 0.023) compared to patients with negative/strong MYC staining. Again, within the group of pRCC type 2 tumors, the MYC staining intensities were not associated with a different risk of death. A multivariate analysis of CSS was not meaningful due to the absence of tumor-related casualties (Table 3).

### Multivariate predictive models

**Figure 4:**
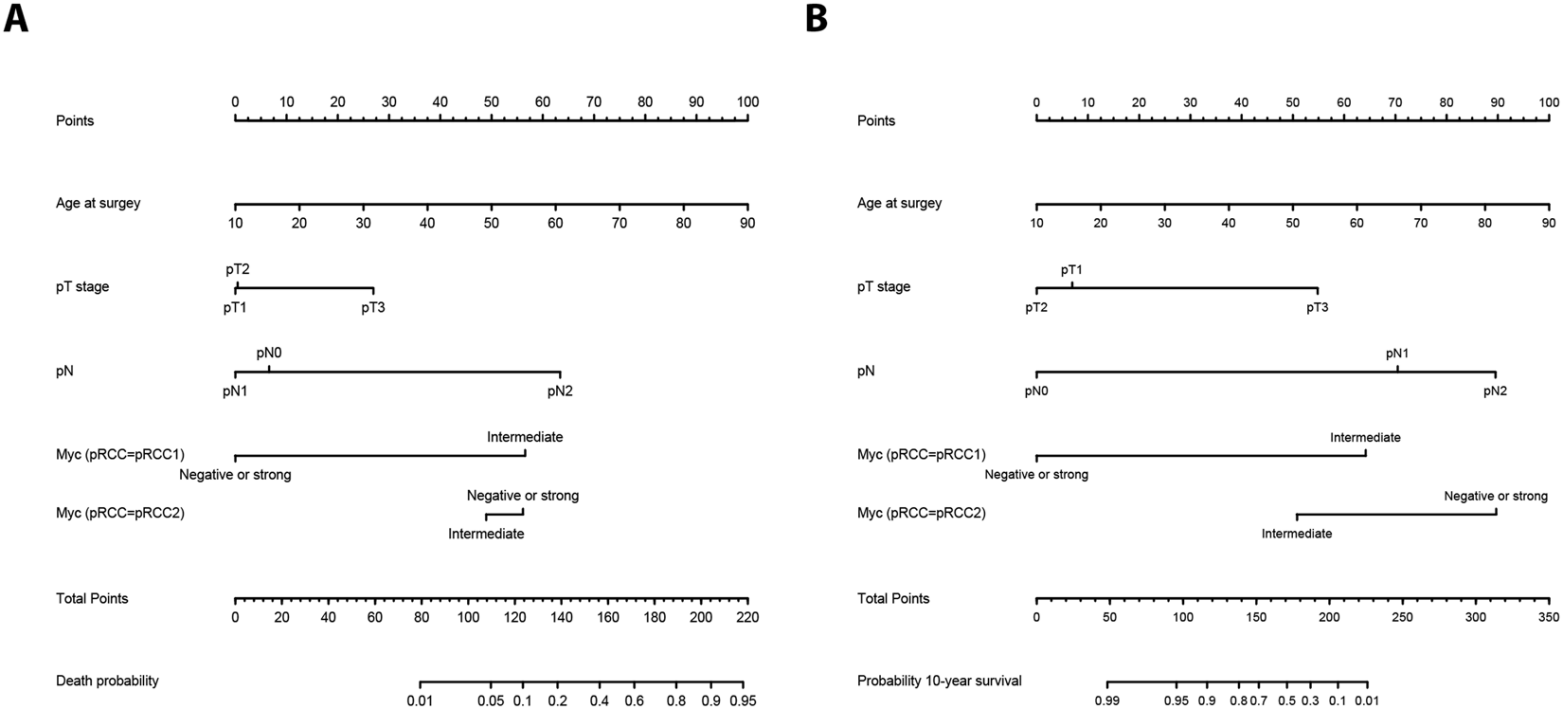
Nomograms predicting the patients’ (**A**) individual probability of death (OS) and (**B**) individual probability of a 10-year overall survival, depending on their age at surgery, the tumor stage, lymph node invasion and the MYC staining patterns within both histological pRCC subtypes.

We next generated regression models to predict an individual’s probability of death (linear regression modeling) and ten-year survival probability (proportional hazard regression modeling). The covariates used in the models were the patient’s age at surgery, pathological tumor stage, presence of lymph node invasion and interaction of the pRCC subtype (pRCC types 1 and 2) with the MYC staining patterns. For visualization, we constructed nomograms to estimate the individual patient’s death probability (Fig. 4A) and ten-year survival probability (Fig. 4B). We discovered that the MYC staining patterns predominantly had an influence in the pRCC type 1 tumors. Obviously, a pRCC type 2 histology is already associated with a high risk of death; therefore, the differences in the MYC staining patterns do not add any prognostic knowledge to this subgroup. The associated bootstrap adjusted calibration plots, which illustrate the accuracy of the generated models, are shown in Supplementary Figs 11 and 12.

## Discussion

The histomorphological identification of subtypes of human cancers has gained increasing importance in stratifying patients and providing more accurate predictions of the clinical course of the disease. Although the histomorphological characteristics of type 1 and type 2 pRCCs have already been described^21^, these subtypes were introduced much later in the 2004 WHO classification system for malignant tumors of the urinary tract^22^. In the 2016 WHO classification, even more histomorphologically or molecularly defined RCC subtypes are listed^2^. Despite the discussion about the practical differentiation between pRCC types 1 and 2 due to the frequent overlap of the morphologies and cases with both type 1 and type 2 morphology, the 2016 WHO classification recommends that the type 1/type 2 pRCC classification system is retained. Regarding pRCC tumors with a mixed (type 1/type 2) histomorphology, one study reported that such mixed pRCC tumors were indistinguishable from pRCC type 1 tumors in terms of cancer-specific survival^18^. However, that study only included five cases of mixed-histology pRCC. Our patient cohort included 34 cases of mixed-histology pRCC, and this patient group exhibited worse overall and cancer-specific survival compared to the pRCC type 2 cases.

We have previously characterized the miRNA expression profiles in the histomorphological pRCC subtypes^12^. We showed that only two miRNAs, *miR-210* and *let-7c*, were sufficient to correctly classify 19 of 22 pRCC samples into type 1 or type 2. A subsequent pathway analysis indicated that several genes of the Jak-STAT signaling pathway, including MYC, might be targeted by the deregulated miRNAs^12^. One recent study^23^ demonstrated that MYC immunoreactivity was not detected in the majority of 45 consecutive pRCCs, while another study showed that MYC is overexpressed in high-grade papillary RCC samples^15^. Another study^24^ reported increased MYC expression in 24 of 25 ccRCC cases compared to the adjacent non-malignant tissue. In our study, we did not detect any significant differences in survival analyses when comparing negative, intermediate and strong MYC staining patterns. During our analysis, however, the Kaplan-Meier tables suggested a tendency for a survival advantage for the patients with negative or strong MYC staining over intermediate MYC staining. Therefore, we decided to combine these two groups and to compare the combination of negative and strong MYC staining (favorable pattern) to intermediate MYC staining (adverse pattern). Many human cancers appear to be associated with or even strictly dependent on activated MYC signaling (reviewed in ref.^25^). However, it has also been described that excessive MYC signaling sensitizes cancer cells to pro-apoptotic stimuli^26^ and that there is a distinct threshold that determines the pro-mitotic or apoptotic function of MYC^20^. The MYC-induced protein MINA53 was not associated with the clinico-pathological characteristics or patient survival. However, the distribution of the staining results agreed with a recent publication describing the MINA53 and Ki67 staining intensities in predominantly clear cell RCCs^19^.

Ki67 has recently been recognized as independent biomarker for RCC recurrence^27^. In the Kaplan-Meier survival analysis, we also detected that Ki67 overexpression (≥5%) was a negative prognostic factor for CSS (p < 0.001).

In the survival analyses, we noted that only the combination of histological pRCC subtype 1 and the MYC staining patterns was able to define a patient group with an excellent prognosis. None of the patients with a pRCC type 1 and the favorable (negative or strong) MYC staining patterns died from tumor-related causes. This identification might be of significant clinical relevance because in our patient cohort, more than 44% of all patients with a pRCC type 1 histology belonged to this excellent prognostic group.

In the prognostic nomograms used to predict the patient’s survival, we demonstrated that the incorporation of the MYC staining patterns only adds prognostic knowledge to the pRCC type 1 tumor subgroup, independent from the patient’s age at surgery, pathological tumor stage, and presence of lymph node invasion. Type 2 histomorphology itself confers a high risk status, which cannot be influenced by the MYC staining patterns. Therefore, MYC staining does not add any prognostic knowledge to this pRCC subtype.

In summary, in a large series of papillary RCCs, we show that in addition to the established histomorphological classification, the immunohistochemical assessment of MYC is able to provide further knowledge about individual patients’ long-term prognosis.

MYC staining is the only parameter that can further sub-stratify pRCC type 1 patients into better and worse prognosis groups. None of the patients with pRCC type 1 tumors and the favorable MYC staining patterns died of tumor-related causes during the complete observation period of 141 months. The MYC staining patterns, particularly for pRCC type 1 tumors, provide additional knowledge that can be used to predict an individual patient’s long-term prognosis.

## Patients and Methods

### Patients

A total of 204 patients with papillary RCC were retrospectively analyzed. The patients’ sample collection was a joint collaboration of the PANZAR consortium. The contributing institutions were (in alphabetical order) Erlangen, Heidelberg, Herne, Homburg, Mainz, Mannheim, Marburg, Muenster, LMU Munich, TU Munich and Regensburg. The participating institutions obtained written informed consent from the patients concerning the analysis of tumor tissue and clinical followup information for scientific purposes. The patients’ sample collection represents the subset of pRCC tumors derived from the previously described collection^28^. The study was conducted using pseudonymized information and performed according to the standards established in the Declaration of Helsinki. Renal surgery was performed between 1993 and 2007. After review by an experienced uropathologist (AH), one representative area of the pRCC tumors was selected to construct the tissue microarrays. For each case, the papillary subtype was defined^2^ and pathological TNM staging was performed^29^. The patients’ clinico-pathological characteristics are presented in Table 1.

### Immunohistochemistry

Briefly, 3 µm slices of the tissue microarray were prepared. After deparaffinization and re-hydration, the target epitopes were unmasked by a 5 minute heat treatment in TE buffer (10 mM Tris, 1 mM EDTA, pH = 8.5) at 120 °C. After blocking the endogenous peroxidase activity with peroxidase blocking solution (Dako, Glostrup, Denmark), the tissue sections were incubated for 1 hour with the following primary antibodies diluted in antibody diluent (Dako): c-MYC, dilution 1:50 (ab32072, clone Y69, Abcam, Cambridge, UK); MINA53, dilution 1:200 (ab126282, Abcam); and Ki67, dilution 1:100 (clone MIB1, Dako). The bound antibodies were visualized with an HRP-conjugated secondary antibody (Dako) and the diaminobenzidine chromogen (Dako).

### Statistics

Comparisons of the continuous variables were conducted using non-parametric Mann-Whitney and Kruskal-Wallis statistical tests and comparisons of the categorical variables were conducted using Chi-squared statistical tests. The differences in the patients’ survival times were examined using the Kaplan-Meier method and log-rank statistics. The mean survival times were calculated using the restricted mean method, which is applicable for highly censored data^30^. The relative risks for a patient’s survival were established by fitting uni- and multivariate Cox's regression models. All calculations were performed with the R statistical framework Ver. 3.2.1 (R Foundation for Statistical Computing, Vienna, Austria. http://www.R-project.org/). The predictive models and prognostic nomograms were constructed using the RMS package for R.

## Electronic supplementary material

Supplementary Information
