# Supplementary material for: Differential prognostic value of MYC immunohistochemistry in subtypes of papillary renal cell carcinoma
Source: Sci Rep. 2017 Nov 27;7:16424. doi: 10.1038/s41598-017-16144-4 (PMC5703709; doi:10.1038/s41598-017-16144-4)
Supplement: Supplementary file 1 — Supplementary Information [file 41598_2017_16144_MOESM1_ESM.doc]

**Differential prognostic value of MYC immunohistochemistry in subtypes of papillary renal cell carcinoma**

Julia Bellut^1,a^, Simone Bertz^2,a^, Elke Nolte^1^, Christine Stöhr^2^, Iris Polifka^2^, Verena Lieb^1^, Edwin Herrmann^3^, Rudolf Jung^2^, Arndt Hartmann^2^, Bernd Wullich^1^, Helge Taubert^1.*^, and Sven Wach^1^

^1^Department of Urology, University Hospital Erlangen, FAU Erlangen-Nürnberg, Germany; ^2^Institute of Pathology, University Hospital Erlangen, FAU Erlangen-Nürnberg, Germany; ^3^University Hospital Münster, Germany

**Supplementary tables and figures.**

| Supplementary table 1. Association of MYC staining patterns with patient characteristics | | | | | |
| --- | --- | --- | --- | --- | --- |
|  |  | MYC negative (N=69) | MYC intermediate (N=113) | MYC strong (N=22) | P |
| Age at surgery; median(IQR) | | 64.25 (56-74) | 63 (54-69) | 66.5 (62.25-75) | 0.139 |
| Gender; N | |  |  |  | 0.949 |
|  | Female | 14 | 24 | 4 |  |
|  | Male | 54 | 89 | 18 |  |
|  | n.a. | 1 | 0 | 0 |  |
| pT stage; N | |  |  |  | 0.640 |
|  | pT1 | 43 | 64 | 10 |  |
|  | pT2 | 13 | 23 | 6 |  |
|  | pT3 | 12 | 25 | 6 |  |
|  | pT4 | 1 | 0 | 0 |  |
|  | n.a. | 0 | 1 | 0 |  |
| pN; N | |  |  |  | 0.226 |
|  | pN0 | 59 | 93 | 19 |  |
|  | pN1 | 5 | 2 | 2 |  |
|  | pN2 | 4 | 11 | 1 |  |
|  | n.a. | 1 | 7 | 0 |  |
| pM; N | |  |  |  | 0.267 |
|  | M0 | 60 | 97 | 22 |  |
|  | M1 | 7 | 12 | 0 |  |
|  | n.a. | 2 | 4 | 0 |  |
| Grade; N | |  |  |  | 0.957 |
|  | G1 | 17 | 28 | 5 |  |
|  | G2 | 40 | 69 | 14 |  |
|  | G3 | 10 | 13 | 2 |  |
|  | n.a. | 2 | 3 | 1 |  |
| Status OS; N | |  |  |  | 0.658 |
|  | Alive | 52 | 81 | 18 |  |
|  | Deceased | 15 | 29 | 4 |  |
|  | n.a. | 2 | 3 |  |  |
| Status CSS; N | |  |  |  | 0.341 |
|  | Other | 58 | 92 | 21 |  |
|  | Cancer specific death | 9 | 18 | 1 |  |
|  | n.a. | 2 | 3 | 0 |  |
| IQR, interquartile range; n.a., not available | | | | | |

| Supplementary table 2. Association of MINA53 staining patterns with patient characteristics | | | | |
| --- | --- | --- | --- | --- |
|  |  | MINA negative (N=133) | MINA positive (N=71) | P |
| Age at surgery; median(IQR) | | 63 (54-70) | 66 (58.22-72.08) | 0.114 |
| Gender; N | |  |  | 0.359 |
|  | Female | 25 | 17 |  |
|  | Male | 108 | 53 |  |
|  | n.a. | 0 | 1 |  |
| pT stage; N | |  |  | 0.545 |
|  | pT1 | 78 | 39 |  |
|  | pT2 | 28 | 14 |  |
|  | pT3 | 27 | 16 |  |
|  | pT4 | 0 | 1 |  |
|  | n.a. | 0 | 1 |  |
| pN; N | |  |  | 0.710 |
|  | pN0 | 111 | 60 |  |
|  | pN1 | 7 | 2 |  |
|  | pN2 | 10 | 6 |  |
|  | n.a. | 5 | 3 |  |
| pM; N | |  |  | 0.790 |
|  | M0 | 117 | 62 |  |
|  | M1 | 13 | 6 |  |
|  | n.a. | 3 | 3 |  |
| Grade; N | |  |  | 0.473 |
|  | G1 | 35 | 15 |  |
|  | G2 | 82 | 41 |  |
|  | G3 | 14 | 11 |  |
|  | n.a. | 2 | 4 |  |
| Status OS; N | |  |  | 0.320 |
|  | Alive | 100 | 51 |  |
|  | Deceased | 28 | 20 |  |
|  | n.a. | 5 | 0 |  |
| Status CSS; N | |  |  | 0.200 |
|  | Other | 113 | 58 |  |
|  | Cancer specific death | 15 | 13 |  |
|  | n.a. | 5 | 0 |  |
| IQR, interquartile range; n.a., not available | | | | |

| Supplementary table 3. Association of the Ki67 labeling index with patient characteristics | | | | |
| --- | --- | --- | --- | --- |
|  |  | Ki67 <5% (N=126) | Ki67 ≥5% (N=71) | P |
| Age at surgery; median(IQR) | | 64 (55.25-71.25) | 63 (53.5-70.5) | 0.612 |
| Gender; N | |  |  | 0.756 |
|  | Female | 27 | 14 |  |
|  | Male | 98 | 57 |  |
|  | n.a. | 1 | 0 |  |
| pT stage; N | |  |  | 0.004 |
|  | pT1 | 76 | 35 |  |
|  | pT2 | 31 | 10 |  |
|  | pT3 | 18 | 25 |  |
|  | pT4 | 1 | 0 |  |
|  | n.a. | 0 | 1 |  |
| pN; N | |  |  | 0.031 |
|  | pN0 | 110 | 54 |  |
|  | pN1 | 4 | 5 |  |
|  | pN2 | 6 | 10 |  |
|  | n.a. | 6 | 2 |  |
| pM; N | |  |  | 0.032 |
|  | M0 | 115 | 57 |  |
|  | M1 | 8 | 11 |  |
|  | n.a. | 3 | 3 |  |
| Grade; N | |  |  | 0.073 |
|  | G1 | 33 | 14 |  |
|  | G2 | 78 | 41 |  |
|  | G3 | 11 | 14 |  |
|  | n.a. | 4 | 2 |  |
| Status OS; N | |  |  | 0.296 |
|  | Alive | 96 | 48 |  |
|  | Deceased | 28 | 20 |  |
|  | n.a. | 2 | 3 |  |
| Status CSS; N | |  |  | 0.002 |
|  | Other | 113 | 51 |  |
|  | Cancer specific death | 11 | 17 |  |
|  | n.a. | 2 | 3 |  |
| IQR, interquartile range; n.a., not available | | | | |

Supplementary Figure 1. Kaplan-Meier estimates of overall survival of patients stratified according to the histological pRCC subtype. The P-value was derived from the log-rank test.

Supplementary Figure 2. Kaplan-Meier estimates of overall survival of patients stratified according to the MYC staining intensity. The P-value was derived from the log-rank test.

Supplementary Figure 3. Kaplan-Meier estimates of tumor specific survival of patients stratified according to the MYC staining intensity. The P-value was derived from the log-rank test.

Supplementary Figure 4. Kaplan-Meier estimates of overall survival of patients stratified according to the MINA53 staining intensity. The P-value was derived from the log-rank test.

Supplementary Figure 5. Kaplan-Meier estimates of tumor specific survival of patients stratified according to the MINA53 staining intensity. The P-value was derived from the log-rank test.

Supplementary Figure 6. Kaplan-Meier estimates of overall survival of patients stratified according to the Ki67 labeling index. The P-value was derived from the log-rank test.

Supplementary Figure 7. Kaplan-Meier estimates of tumor specific survival of patients stratified according to the Ki67 labeling index. The P-value was derived from the log-rank test.

Supplementary Figure 8. Kaplan-Meier estimates of overall survival of patients stratified according to the combination of histologic pRCC subtype and the MYC staining intensity. The P-value was derived from the log-rank test.

Supplementary Figure 9. Kaplan-Meier estimates of overall survival of patients stratified according to the combination of histologic pRCC subtype and the MINA53 staining intensity. The P-value was derived from the log-rank test.

Supplementary Figure 10. Kaplan-Meier estimates of overall survival of patients stratified according to the combination of histologic pRCC subtype and the Ki67 labeling index. The P-value was derived from the log-rank test.

Supplementary figure 11. Calibration plot for nomogram predicting patients’ individual risk of death in dependence of the age at surgery, tumor stage, lymph node invasion and the MYC staining patterns within both histological pRCC subtypes based on 100 bootstrap repetitions.

Supplementary figure 12: Calibration plot for nomogram predicting patients’ individual probability of a 10-year survival in dependence of the age at surgery, tumor stage, lymph node invasion and the MYC staining patterns within both histological pRCC subtypes based on 100 bootstrap replications.
